# Designing, implementing, and evaluating a mobile app-based cultural care training program to improve the cultural capacity and humility of nursing students

**DOI:** 10.1186/s12909-023-04952-4

**Published:** 2023-12-20

**Authors:** Sara Noori Farsangi, Sedigheh Khodabandeh Shahraki, Jonas Preposi Cruz, Jamileh Farokhzadian

**Affiliations:** 1 Reproductive Health, Family and Population Research Center, Kerman University of Medical Sciences, Kerman, Iran; 2 Health in Disasters and Emergencies Research Center, Institute for Futures Studies in Health, Kerman University of Medical Sciences, Kerman, Iran; 3 Department of Medicine, School of Medicine, Nazarbayev University, Astana, Kazakhstan; 4 Nursing Research Center, Kerman University of Medical Science, Kerman, Iran

**Keywords:** Cultural care, Cultural competence, Cultural capacity, Cultural humility, Nursing students, Mobile application

## Abstract

**Background:**

Given the growing cultural diversity among healthcare clients, it is crucial for nursing education to have a clear mission: to effectively train future nurses by incorporating cultural care curricula. The goal is to equip them with the necessary cultural capacity and humility. This study focused on designing, developing, and evaluating a mobile app-based cultural care training program, with the aim of enhancing the cultural capacity and humility of nursing students.

**Methods:**

This experimental study utilized the five steps of the ADDIE instructional model (analysis, design, development, implementation, and evaluation) to design a mobile app-based cultural care training program. The first three steps involved designing and developing the program, drawing upon Purnell's model for cultural competence and Foronda's rainbow model of cultural humility. In the fourth step, the cultural care training program was implemented in 16 modules among 80 internship nursing students. These students were randomly assigned to either the intervention or control groups, with 40 students in each group. Finally, in the fifth step, the effectiveness of the mobile app-based program was evaluated by administering the Cultural Capacity Scale, and the Foronda Cultural Humility Scale before and one month after the cultural care training. The collected data were analyzed using SPSS22, employing techniques such as paired t-test, chi-square test, and independent samples t-test.

**Results:**

A total of 76 students completed the study, with 39 students in the intervention group and 37 students in the control group. Prior to the mobile app-based cultural care training program, there were no significant differences in cultural capacity and humility scores between the two groups (*p* > 0.05). However, following the completion of the program, the intervention group exhibited higher scores in cultural capacity and humility compared to the control group (*p* < 0.05).

**Conclusion:**

Based on the findings, it can be concluded that the mobile app-based cultural care training program had a positive impact on the cultural capacity and humility of undergraduate nursing students. These results indicate the importance of nurse educators designing comprehensive training programs that incorporate innovative approaches to enhance cultural capacity and humility among nursing students at all academic levels.

## Introduction

The world is currently witnessing a continuous increase in global migration. Globalization, epidemiological conditions, social conflicts, and natural disasters frequently contribute to increased migration patterns. Working within a culturally diverse environment imposes a moral and professional obligation on nursing students, nurses, their organizations, educational institutions, professional associations, and the healthcare system to deliver culturally competent or culturally sensitive care [1].

The purpose and mission of cultural care are centered around investigating, understanding, and explaining the reciprocal relationship between nursing care and culture [2]. Cultural care is grounded in a humanistic and holistic approach that integrates qualities such as compassion, honesty, kindness, and altruism in the provision of healthcare, while also considering the cultural needs of patients [3]. In order to deliver effective cultural care, it is essential to develop cultural competence and capacity. Cultural competence is defined as embracing open-mindedness and showing respect for individuals, families, and communities from diverse cultural backgrounds. The ability to apply this cultural knowledge and these skills in practice can contribute to improving the cultural appropriateness of healthcare [4]. It encompasses a range of behaviors, attitudes, and policies that are congruent and facilitates effective communication in cross-cultural situations [5].

Cultural competence should be incorporated into the nursing curriculum. The absence of cultural care training in nursing education can have adverse effects. For instance, nurses may resort to personal experiences to address the cultural needs of patients, potentially compromising their cultural safety. Furthermore, the absence of cultural competence can result in prejudice, discrimination, and miscommunication among culturally diverse individuals [6].

Nursing education has long recognized that cultural competence should be a key outcome of baccalaureate programs. The American Association of Colleges of Nursing has offered a comprehensive framework of learning expectations and strategies to guide nursing educators in preparing culturally competent nursing students [5]. Unfortunately, cultural competence and capacity are not given sufficient emphasis in nursing education, leaving students feeling ill-prepared to provide culturally competent care to diverse populations. Students may struggle with confidence, transcultural self-efficacy, and language barriers when assessing patients from different cultural backgrounds, hindering their ability to provide effective cultural care [3].

The literature review reveals that nursing students in various countries exhibit a low to moderate level of cultural competence, and cultural competence has been overlooked in nursing education [5–11]. Almutairi et al. (2017) demonstrated that factors such as age, work experience, and birthplace influenced nurses’ perception of cultural competence. They concluded that developing cultural competence required exposure to diverse patient populations and the acquisition of cultural knowledge and awareness [12]. Other studies have reported that several factors, including lack of cultural diversity education, absence of ethnic role models, racial discrimination, lack of support [3], neglect of cultural competence in educational approaches, inattention to cultural care in clinical settings, poor intercultural communication, and insufficient skill in cultural humility, have had negative effects on students’ cultural competence. Researchers have emphasized the need to enhance nursing students’ cultural competence through cultural care education [13]. In addition, several studies in Iran highlighted that the presence of a multicultural and multi-ethnic environment, along with factors such as changing migration patterns, international staff recruitment [14], the rise of medical tourism, and the influx of culturally diverse patients [15] necessitates the development of cultural capacities and competencies among nurses, nursing students, and nursing professors. This is crucial for enhancing the medical tourism industry and delivering culturally congruent care to a diverse range of clients [16].

In addition to cultural competence and capacity, cultural humility is crucial for nurses and nursing students to provide comprehensive and individualized cultural care. Cultural humility involves recognizing diversity and power imbalances, being open, self-aware, flexible, and respectful, and focusing on both oneself and others to develop tailored responses. It is a process of self-reflection and lifelong learning that leads to positive outcomes [17]. Without cultural humility, healthcare providers may assume that everyone shares their worldview, leading to cultural oppression and the imposition of cultural values on diverse clients and communities. Understanding the history, life experiences, cultural values, and aspirations of different cultural groups is essential for healthcare professionals to be competent providers [18].

The literature review indicates that there have been limited studies conducted in various countries [3, 5, 19] and specifically in Iran [16, 20] to evaluate the effect of cultural care training programs on the cultural competence of nursing students. These studies utilized a combination of face-to-face and online methods for training. Several researchers have attempted to develop educational programs aimed at cultivating the cultural competence and humility of nurses and nursing students by utilizing cultural competence and humility models [17, 21–24]. The findings from these studies revealed that nursing students generally lacked sufficient cultural competence before the training. However, after completing the training, participants in the intervention group demonstrated significant improvements in their cultural competence.

A systematic review highlighted the significant progress made in nursing education through the integration of wireless internet and mobile technologies [25]. While there has been an increase in the number of mobile learning studies in nursing education, only one intervention study was found that utilized a mobile application to teach cultural competence. This study developed and evaluated a cultural competence training program for nurses using a mobile application. The researchers concluded that mobile-based learning, with its collaborative nature, high portability, responsiveness, increased learner participation, and cost-effectiveness, can serve as a suitable alternative to printed materials in nursing education [21].

Given the process of globalization and the increasing cultural diversity among healthcare users, new demands and challenges are emerging for healthcare professionals in various countries, particularly for nursing students who will be working in dynamic and globally connected work environments in the future. Understanding and developing cultural competence, capacity, and humility are crucial factors in effectively addressing the diverse needs of patients and fostering trusting relationships between clients and health professionals [1]. Furthermore, the aforementioned studies have indicated that nurses acquire cultural competence, capacity, and humility from personal experiences in practice and during their academic studies. Nursing education has a responsibility to prepare future nurses to attain cultural competence and capacity and to deliver cultural care with humility to diverse clients. Therefore, their cultural competence and capacity should be assessed, and educational interventions should be implemented to develop and enhance their cultural capacity, competence, and humility. Additionally, existing technologies and modern communication methods such as mobile applications can enhance student learning in an engaging manner. However, there is a dearth of studies focusing on cultural capacity and humility, particularly in relation to modern educational interventions such as the use of mobile applications and cultural competence and humility models. This study aimed to design, develop, and assess a mobile app-based cultural care training program to enhance the cultural capacity and humility of nursing students.

## Methods

### Study design

This experimental research followed a pre-test, post-test design and was conducted at two comprehensive urban health centers affiliated with the Kerman University of Medical Sciences in southeastern Iran. The study included two groups: an intervention group and a control group.

According to the academic regulations in Iran, a bachelor's degree in nursing typically consists of four years of study, which includes both theoretical and clinical coursework. During the final year of the program, nursing students participate in an internship program that aims to prepare them for professional nursing practice. During this internship, senior students work under the supervision of nurse instructors in various clinical settings. To ensure effective clinical education and address any educational challenges faced by the students, the school of nursing appoints faculty members from each educational department based on the students' credits. In Iran, cultural competence, capacity, and humility have received minimal attention in the nursing education program and are not formally addressed in the official curriculum. Students continue to learn about cultural care through an informal program. Similarly, nurses acquire cultural competence and humility through their work experience in healthcare settings [26].

In this study, a mobile app-based cultural care training program was developed specifically for senior undergraduate nursing students. The development process followed the five stages of the ADDIE instructional model, which included analysis, design, development, implementation, and evaluation.

#### Step 1: analysis

The primary objective of the mobile app-based cultural care training program was to enhance the cultural capacity and humility of nursing students. During the analysis stage, specific objectives were identified, along with the necessary features of audiences, resources, and technology required for the program. Additionally, methods for presenting the content and ensuring the performance of the mobile application were determined. To guide the design and development of the program's content, Purnell's model for cultural competence and Foronda's rainbow model of cultural humility were selected as the framework [17, 27].

#### Step 2: design

During the design stage, the learning modules, content, and educational strategies for the mobile application were determined. The educational content was developed following a review of the literature [6, 10, 14, 17, 21, 22, 26–29] and was presented in the form of 16 modules in the Persian language. These modules included one general module covering cultural care and an overview of the Purnell model, 12 modules focusing on the cultural domains of the Purnell model, and 3 modules based on Foronda’s rainbow model of cultural humility. Additionally, the app included 8 videos and 4 images. The images included visuals such as the culture iceberg, Purnell's model for cultural competence, and the rainbow model of cultural humility. The references of the text used in the app were cited separately in the references section. The mobile app was designed to operate on Android 4.4 or higher.

To ensure the quality and accuracy of the module contents, validation was conducted by three faculty members from the Razi School of Nursing and Midwifery. The cultural care training curriculum was thoroughly reviewed and revised based on their feedback (Table 1).

**Table 1:** Themes covered in the training curriculum

Modules	Main topics	Contents
1	Introduction: Concepts of cultural capacity, competence, and cultural care	- Culture, cultural diversity, and components of cultural diversity Cultural competence and cultural care An overview of Purnell's cultural competence model and the 12 domains of the cultural competence model Assessment of patients from different cultural backgrounds Planning for cultural nursing care
2	Domain 1: Culture and heritage	- Culture and heritage Concepts related to the country of origin, current residence, economics, political science Reasons for migration, and educational and professional status Assessing the client according to this field
3	Domain 2: Communication	-The domain of communication Cultural communication patterns Verbal and non-verbal interactions (eye contact, facial expression, and touch) The use of language, dialect, use of hand and face gestures in addition to oral speech, which are distinct in each cultural group The use of an interpreter Assessing the client according to this field
4	Domain 3: Family roles and organization	- Views related to the head of the household, gender roles, goals, and priorities, Duties and roles of the family The roles of children, adolescents, older people, and the extended family Social position, alternative lifestyles Assessing the client according to this field
5	Domain 4: Labor issues	- Cultural acceptance, independence, and the presence of language barriers) Assessing the client according to this field
6	Domain 5: bio-cultural ecological factors	- Observable differences according to ethnic and racial origin such as skin color and other physical, biological, and physiological changes among racial and ethnic groups Assessing the client according to this field
7	Domain 6: Risky behaviors	- High-risk behaviors (including the use of tobacco, alcohol, or dangerous drugs, smoking, sexual relations, high-fat diets, and physical inactivity) Physical activities and safety levels or precautions Assessing the client according to this field
8	Domain 7: Nutrition	- Having enough food, the value of food Common foods and customs Food restrictions and nutritional deficiencies The use of food to promote health and prevent diseases Assessing the client according to this field
9	Domain 8: Pregnancy	- Fertility practices, views, and beliefs regarding childbearing, birth, and postpartum pregnancies Forbidden reproductive functions, child custody, parenting, prescriptive practices Food recommendations, restrictions, and taboos Assessing the client according to this field
10	Domain 9: Death rituals	- Mourning ceremony Perceptions of death, acceptance of death, death ceremony Functions and views on death and mourning, and beliefs about life after death Assessing the client according to this field
11	Domain 10: Spirituality	- Spirituality and religious practices, use of prayer, individual strength Meaning of life and the relationship between spirituality and healthcare functions Assessing the client according to this field
12	Domain 11: Healthcare practices	- Common traditional practices (e.g., complementary and alternative medicine), self-healing practices, magical religious beliefs, responsibility for cultural barriers to healthcare, cultural responses to health and illness Response to pain, treatment and rehabilitation, chronic disease, mental health measures, blood transfusion, and organ donation Assessing the client according to this field
13	Domain 12: Healthcare providers	-Practices of healthcare providers Traditional care versus biomedical care The status of healthcare providers (for example, their expertise and qualifications, their willingness to work in a multicultural society, their ethnic and racial status, and their gender), The role of traditional religious care providers, folk, and sorcerers and their impact on health providers) Assessing the client according to this field
14	Cultural humility	-The definition of cultural humility The basics of cultural humility The rainbow model of cultural humility
15	Necessary skills for cultural humility	-Skills necessary for cultural humility (reflection, respect, regard, relevance, resiliency) and their definitions
16	Consequences of cultural humility	-Consequences of cultural humility (learning from patients and empowerment, partnership-building, lifelong learning process) and their definitions

#### Step 3: development

During the development stage, a mobile application called the cultural care training program was designed and created by SPERLOS, as outlined in Table 1. The app was developed to offer students comprehensive and easily understandable educational content across various categories, employing visual effects and practical training methods. The app development involved creating 37 screens, including pages for 16 learning modules, settings, references, and goals, incorporating text, 8 videos, and 4 images aligned with the module content. Additionally, a formative test based on the module content was integrated. The materials were arranged on the pages to enable students to dedicate an average of 10 to 15 min to each module. The intention behind this organization was to ensure that students could study the materials efficiently without feeling overwhelmed or bored.

A heuristic evaluation of the mobile app was conducted by three medical informatics specialists to assess its usability and user-friendliness. Following the evaluation, a pilot test was carried out to determine the comprehensibility of all the modules before implementing the training program. For the pilot test, four nursing students were selected as participants, and no revisions were requested.

#### Step 4: implementation

During this phase, the sample size was determined according to previous studies [10, 16] and the sample size formula. With α = 0.05, a test power of 80%, and a moderate effect size (Cohen d = 0.6), the required sample size was calculated to be 35 participants for each group. To improve the results and increase the statistical power of the test while accounting for dropout probability, the required sample size was adjusted to 40 participants in each group (resulting in a total of 80 participants for both groups). The study population included all senior nursing students from the School of Nursing and Midwifery (*N* = 80) at the time of data collection. Given that the sample size was equal to the study population, all students were included in the study by a census. Subsequently, the students were assigned into the intervention (*n* = 40) and control (*n* = 40) groups using a random number table. The inclusion criteria for the study students included passing the Objective Structured Clinical Examination (OSCE) for the nursing internship course, being willing to participate in the training program, owning a smartphone, and having the ability to use a mobile-based application. Exclusion criteria consisted of unwillingness to install the app on their mobile phones at the designated time, departure or transfer to another faculty, and failure to complete more than ten percent of the questions in questionnaires.

After the random assignment of students to the intervention and control groups, the project manager held separate meetings with the students from each group. During these meetings, the project manager explained the objectives and procedures of the study and obtained written consent from the students to participate in the study. Following the consent process, pre-test data were collected from both the intervention and control groups. This data collection involved the administration of a demographic and professional information questionnaire, the Cultural Capacity Scale, and the Foronda Cultural Humility Scale, which were provided in paper form. Before commencing their activities at the centers, the students from both the control and intervention groups were divided into smaller groups of four individuals each. Based on the predetermined schedule, a total of 8 students (4 from each group) attended the comprehensive health centers every month to undertake “community, family, and individual nursing internships”. The duration of each group's internship period was one month. To ensure fairness, one center was designated as the intervention group and the other as the control group each month, with the centers being switched the following month. The entire process of the intervention, spanning from September 23, 2022, to July 22, 2023, covered a period of ten months.

To execute the training program, the first researcher visited the comprehensive health centers at the onset of each month in line with the students’ internship program. The researcher presented the students with an overview of the educational objectives and the mobile application for the cultural care training program. She provided instructions to the students in intervention group on how to register and install the cultural care training application on their mobile phones. Additionally, the researcher explained how to utilize the program at scheduled times and complete evaluations. The students’ contact numbers were also collected for follow-up purposes. To ensure their active participation, the first author sent reminder messages to the participants’ mobile phones every three days, prompting them to engage with the application’s content. In order to avoid the spread of treatment effects between the intervention and control groups, the members of the intervention group were advised not to share educational content with the control group until the study was completed.

**Fig. 1:**
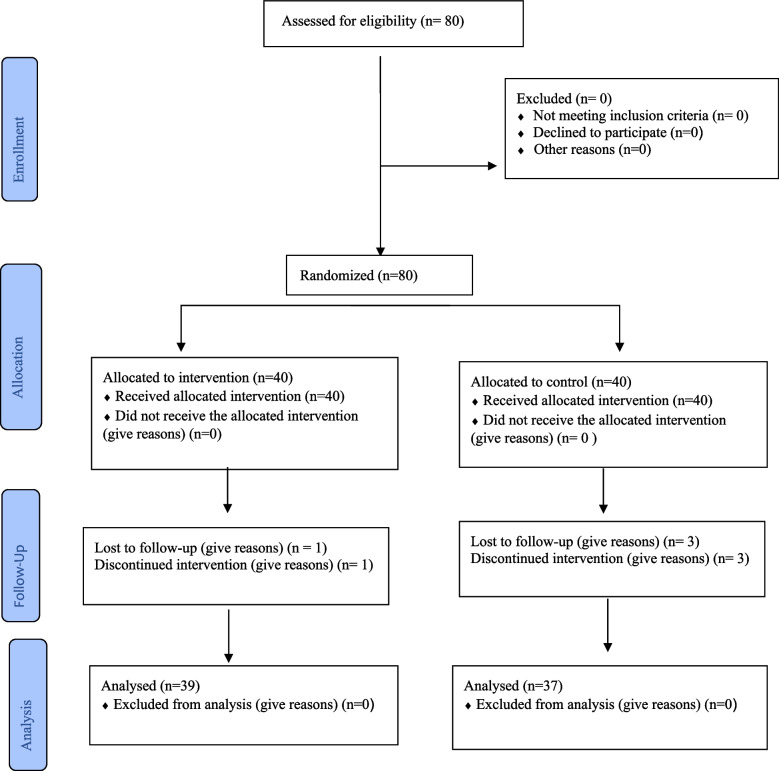
Flow diagram of the study, representing data collection points for the intervention group and the comparison group

At the end of the study, a total of 39 participants from the intervention group and 37 participants from the control group completed the questionnaires. One individual from the intervention group was excluded from the study due to failure to install the mobile application, while 3 individuals from the control group were excluded for not completing the post-test questionnaires (Fig. 1).

#### Step 5: evaluation

The evaluation phase of the study consisted of both formative and summative evaluations. During the formative evaluation, the intervention group was required to complete a 15-item test within the software at the end of each month. Out of the 39 students in the intervention group, only 4 students did not achieve the minimum passing score. However, these students were given an additional 7 days to review and study the program. The first researcher closely monitored the outcomes of the formative evaluation and sent alarm messages to remind participants to complete steps 4 and 5 in the program. For the summative evaluation, aimed at assessing the effectiveness of the application, data from both the intervention and control groups were collected one month after the intervention. Three scales were utilized for this purpose: the characteristics questionnaire, the scale of cultural capacity and humility, and the pre-and post-test scores. By comparing these scores, the impact of the intervention could be determined.

Three tools were used to collect data in the study:

1. Demographic and professional information was used to gather information about gender, marital status, ethnicity, history of participating in cultural care training courses, and the level of care for people with cultural diversity in the clinical settings from the participants (Table 2).

**Table 2:** Comparison of demographic and professional information between the two study groups

Variables	Categories	Intervention		Control		χ^2^	*p*- value
		n	%	n	%		
Gender	Male	13	33.3	11	29.7	0.11	0.73
	Female	26	66.7	26	70.3		
Marital status	Single	32	82.1	28	75.7	0.46	0.49
	Married	7	17.9	9	24.3		
Ethnicity	Native of Kerman province	34	87.2	26	70.3	8.29	0.08
	Fars	3	7.7	8	21.6		
	Turkish	1	2.6	0	0		
	Lor	1	2.6	0	0		
	Others	0	0	3	8.1		
History of participating in cultural care training courses	Yes	11	28.2	7	18.9	0.90	0.34
	No	28	71.8	30	81.1		
The level of care for people with cultural diversity in the clinical settings	Low	4	10.3	5	13.5	3.13	0.37
	Moderate	21	53.8	22	59.5		
	High	11	28.2	10	27		
	Very high	3	7.7	0	0		
Age		M ± SD		M ± SD		Independent t- test	*P*-value
		22.16 ± 2.4		22.08 ± 1.13		1.22	0.22

2) The Cultural Capacity Scale Arabic (CCS-A) gathered data for the cultural capacity of the students. This scale was developed by Perng and Watson (2012) [30] and was adapted to the Arabic language by Cruz et al. (2016). CCS-A consists of 20 items divided into three conceptual subscales: cultural knowledge (items 3, 9, 11, 12, 13, 15), cultural sensitivity (items 17, 20), and cultural skills (items 1, 2, 4, 5, 6, 7, 8, 10, 14, 16, 18, 19) [31]. The items in the CCS-A are designed on a 5-point Likert scale, with response options ranging from 1 (never or not at all sure) to 5 (always or completely sure). The total score ranges from 20 to 100, with higher scores indicating greater cultural capacity [32].

The original CCS-A underwent content validity assessment by five experts. The Item Content Validity Index (I-CVI) was found to be 1, indicating unanimous agreement among the experts. The Scale Content Validity Index (S-CVI) was also 1, indicating high content validity. To assess the reliability of the tool, internal consistency and intraclass correlation coefficient (ICC) were calculated. The Cronbach's alpha coefficient was calculated for the entire scale and yielded a value of 0.96. Additionally, the calculated ICC rate was 0.88, indicating good reliability [32].

In this study, permission was obtained to use the Persian version of the cultural capacity scale which underwent a process of cultural adaptation using the translation-back translation method. The reliability of the questionnaire was assessed using internal consistency on a sample of 30 participants. The Cronbach's alpha coefficient was calculated for each subscale, resulting in values of 0.86 for cultural knowledge, 0.87 for cultural sensitivity, 0.91 for cultural skills, and 0.94 for the entire scale.

3) The Foronda’s Cultural Humility Scale, developed by Foronda et al. (2021), consists of 19 questions divided into three subscales:
  1. The Context for Differences in Perspective (questions 1–7): This subscale assesses participants' awareness of various factors that can influence changes in perspective. These factors include the physical environment, historical background, political climate, power imbalance, and situational context.
  2. Self-attributes (questions 8–11): This subscale measures the degree of flexibility, openness, and awareness of cultural prejudices and humility within oneself.
  3. Outcomes of Cultural Humility (questions 12–19): This subscale explores the outcomes of practicing cultural humility such as mutual empowerment, respect, collaboration, partnership, exceptional care, and lifelong learning.

The scoring method for the Foronda Cultural Humility Scale is based on a 5-point Likert scale, ranging from 1 (never/rarely) to 5 (always). The total score ranges from 19 to 95, with higher scores indicating higher levels of cultural humility. Interpreting the scores, a range of 19 to 35 suggests that individuals rarely exhibit cultural humility. Scores between 36 and 75 indicate that individuals sometimes demonstrate cultural humility. A score of 76 to 85 suggests that individuals usually exhibit cultural humility, while a score of 86 to 95 indicates that individuals are habitually culturally humble [17].

The content validity of the Foronda Cultural Humility Scale was assessed by 6 experts. The Item Content Validity Index (I-CVI) exceeded 0.83, indicating a high level of agreement among the experts. The Scale Content Validity Index (S-CVI) was calculated to be 0.96, and the calculated Cronbach's alpha coefficient was 0.85, indicating good internal consistency [33].

In this study, permission was obtained to use the Persian version of the cultural capacity scale, which underwent a process of cultural adaptation using the translation-back translation method. The reliability of the questionnaire was assessed using internal consistency and Cronbach's alpha coefficient on a sample of 30 participants for both the subscales and the entire scale. The Cronbach's alpha coefficient was calculated for the subscales of context for difference in perspective (0.85), self-attributes (0.82), outcomes of cultural humility (0.86), and the entire scale (0.85).

### Statistical analysis

The data from the summative evaluation were analyzed using SPSS22. Descriptive statistics such as frequency, percentage, mean, and standard deviation were utilized to summarize the data. Inferential statistics, including independent samples t-test, paired t-test, and chi-square test, were employed to examine relationships and differences within the data. To determine whether the data followed a normal distribution, the Kolmogorov–Smirnov test was conducted. The significance level for the statistical tests was set at ≤ 0.05.

## Results

The study used the ADDIE instructional model, which consists of five steps: analysis, design, development, implementation, and evaluation. These steps were employed to design, develop, implement, and evaluate a mobile app-based cultural care training program with the goal of enhancing the cultural capacity and humility of nursing students. During steps 1, 2, and 3 in the ADDIE model, a total of 16 learning modules were created. These modules were based on key concepts related to cultural capacity and competence, cultural care, the Purnell model for cultural competence, and Foronda's rainbow model of cultural humility (Table 1). Additionally, the mobile app included 8 videos, and 4 images. In steps 4 and 5, participants were divided into groups and assigned to complete the learning modules over the course of one month. At the end of each month, participants underwent a formative evaluation by answering a 15-question test that assessed their understanding of the modules content. After completing the course, participants were given the opportunity to revisit the learning modules for an additional month. Furthermore, the researchers evaluated the effectiveness of the curriculum by using cultural capacity and humility scales and comparing the participants' pre-test and post-test scores. Table 2 provides demographic and professional information of the nursing students involved in steps 4 and 5. Tables 3 and 4 present a comparison of the changes in cultural capacity and humility scores before and after the intervention for both groups.

**Table 3:** Comparison of cultural capacity scores between control and intervention groups before and after the training program

	Time	Pre-intervention	Post-intervention				
Variables	Groups	M ± SD	M ± SD	Mean difference	ES^*^ (Cohen d)	Paired *t*-test	*P*- value
Cultural knowledge	Intervention	15.48 ± 4.66	18.46 ± 5.51	2.97	0.58	-3.51	**0.001**
	Control	15.24 ± 2.96	15.83 ± 4.69	0.59	0.15	-0.79	0.43
	Independent *t-*test	0.27	2.22				
	*P*-value	0.78	**0.02**				
	ES^*^ (Cohen d)	0.06	0.51				
Cultural sensitivity	Intervention	5.12 ± 1.6	6.58 ± 1.75	1.46	0.87	-4.76	**0.001**
	Control	5.62 ± 1.55	5.91 ± 1.7	0.29	0.31	-0.94	0.35
	Independent *t-*test	-1.36	1.68				
	*P*-value	0.17	0.09				
	ES^*^ (Cohen d)	0.31	0.31				
Cultural skills	Intervention	32.38 ± 8.82	37.69 ± 9.89	5.30	0.56	-3.41	**0.002**
	Control	32.37 ± 5.95	33.89 ± 9.24	1.51	0.19	-1.20	0.23
	Independent *t-*test	0.004	1.72				
	*P*-value	0.99	0.08				
	ES^*^ (Cohen d)	0.001	0.39				
Total cultural capacity	Intervention	53 ± 14.52	62.74 ± 16.68	9.74	0.62	-3.78	**0.001**
	Control	53.24 ± 9.16	55.64 ± 13.46	2.40	0.20	-1.37	0.17
	Independent *t-*test	-0.08	2.03				
	*P*-value	0.93	**0.04**				
	ES^*^ (Cohen d)	0.01	0.46				

^*^Bold *p*-values are significant at a level of ≤ 0.05

Effect size (ES): 0–0.2 = small effect, 0.2–0.5 = moderate effect, > 0.5–0.7 = large effect, and > 0.7 = very large effect

**Table 4:** Comparison of cultural humility scores between control and intervention groups before and after the training program

Variables	Time	Pre-intervention	Post-intervention				
	Groups	M ± SD	M ± SD	Mean difference	ES^*^ (Cohen d)	Paired *t*-test	*P*- value
The context for differences in perspective	Intervention	19.23 ± 5.98	25.02 ± 6.84	5.79	0.90	-5.64	**0.001**
	Control	20.43 ± 6.47	21.02 ± 5.71	0.09	0.5	-0.52	0.60
	Independent *t-*test	-0.84	2.75				
	*P*-value	0.40	**0.007**				
	ES^*^ (Cohen d)	0.19	0.63				
Self-attributes	Intervention	13.66 ± 2.69	15.05 ± 2.86	1.38	0.50	-3.15	**0.003**
	Control	14.29 ± 2.51	14.94 ± 2.68	0.64	0.25	-1.35	0.18
	Independent *t-*test	-1.05	0.16				
	*P*-value	0.29	0.86				
	ES^*^ (Cohen d)	0.24	0.03				
Outcomes of cultural humility	Intervention	30.94 ± 4.76	33.66 ± 4.66	2.71	0.57	-3.54	**0.001**
	Control	32.89 ± 4.56	32.86 ± 5.13	0.02	0.006	0.03	0.97
	Independent *t-*test	-1.18	0.71				
	*P*-value	0.07	0.47				
	ES^*^ (Cohen d)	0.41	0.16				
Total of cultural humility	Intervention	63.84 ± 9.93	73.74 ± 10.73	9.89	0.95	-6.98	**0.001**
	Control	67.62 ± 8.77	68.83 ± 9.47	1.21	0.13	-0.81	0.41
	Independent *t-*test	-1.73	2.10				
	*P*-value	0.08	**0.03**				
	ES^*^ (Cohen d)	0.41	0.48				

^*^Bold *p*-values are significant at a level of ≤ 0.05

Effect size (ES): 0–0.2 = small effect, 0.2–0.5 = moderate effect, > 0.5–0.7 = large effect, and > 0.7 = very large effect

### Demographic and professional information of nursing students

The study included a total of 76 nursing students, with a response rate of 95%. The mean ages of the nursing students in the intervention and control groups were 22.16 and 22.08 years, respectively. The majority of nursing students in both groups were female (66.7% in the intervention group and 70.3% in the control group), single (82.1% in the intervention group and 75.5% in the control group), and native of Kerman province (87.2% in the intervention group and 70.3% in the control group). Most of the students in both groups had not previously participated in cultural care training courses (71.8% in the intervention group and 81.1% in the control group). Additionally, the majority of students in both groups provided care for culturally diverse people in clinical settings at moderate levels (53.8% in the intervention group and 59.5% in the control group). The information suggests that the two study groups were similar in terms of demographic and professional characteristics (Table 2).

### Outcomes

#### Comparison of changes in cultural capacity

Based on the results presented in Table 3, the study examined the total cultural capacity score and scores for three conceptual subscales of the Cultural Capacity Scale in both the intervention and control groups before and after the training program. Before the training program, there was no significant difference in the score of cultural capacity between the intervention group (53.29 ± 14.52) and the control group (53.24 ± 9.16), (t = -0.08, *p* = 0.93).

However, after the training program, the intervention group showed a significant increase in the score of cultural capacity (62.74 ± 13.46) compared to the control group (55.64 ± 13.46). The effect size of this difference was small (Cohen's d = 0.46, t = 2.03, *p* = 0.04).

Furthermore, when comparing the scores within the intervention group before and after the training program, there was an improvement in the total scores of CCS-A and its subscales. The effect sizes for these improvements ranged from 0.56 to 0.87, indicating a moderate to large effect.

#### Comparison of changes in cultural humility

Table 4 illustrates the total score of cultural humility and scores for three subscales in both groups before and after the training program. The between-group comparisons indicated no significant difference in the cultural humility score between the intervention (63.84 ± 9.93) and control groups (67.62 ± 8.77) before the training program. According to the scoring interpretation, both groups exhibited sometimes culturally humble (t = -1.73, *p* = 0.08). The intervention group demonstrated a significant increase in cultural humility scores (73.74 ± 10.73) compared to the control group (68.83 ± 9.47) after the training program. This indicates that the students' cultural humility improved, although they still displayed sometimes being culturally humble, albeit with a small effect size (Cohen d = 0.48, t = 2.10, *p* = 0.03). According to the scoring criteria, scores ranging from 36 to 75 represented sometimes culturally humble.

The within-group comparisons showed that the total scores of cultural humility and its subscales improved in the intervention group after the training program compared to before training. The effect sizes ranged from 0.50 to 0.95, indicating moderate to large effects. According to the scale scoring, the intervention group still displayed sometimes being culturally humble.

## Discussion

This study aimed to develop and evaluate a mobile app-based cultural care training program specifically designed for nursing students in Iran. The study results indicated that the implementation of this program led to significant improvements in cultural capacity and humility among the nursing students.

First, to develop the training program, the researchers followed the five stages of the ADDIE instructional model. This systematic instructional model proved to be effective in guiding the development process of the mobile app-based cultural care training program. In contrast, previous studies that developed cultural care training programs did not adhere to any instructional models and often lacked reporting on their development processes [5, 16, 26, 34–36].

One notable contribution of our study is the provision of detailed information about the curriculum development process. This ensures that nurse educators and other healthcare providers can easily follow the program and adapt it to different cultural contexts. Additionally, the mobile app-based format of the training program allows for convenient in-service training for healthcare providers in various cultures and settings. The utilization of instructional design models is crucial for curricular designers as it enables them to make informed decisions about their designs. Instructional models provide a systematic and reflective process through which instructional principles are applied to develop effective teaching and learning programs. This involves differentiating materials, activities, resources, and evaluation methods. By employing instructional design models, designers gain knowledge about learning theories, conduct systematic learner analysis, employ management techniques, utilize information technology efficiently, and evaluate the teaching and learning process. These models guide designers in constructing instructional programs that consider the needs of learners, objectives, learning outcomes, teaching methods, and evaluation strategies. While there are various instructional design models available, most of them incorporate the essential phases of the ADDIE model. Designers should select instructional design models based on specific learning situations and tailor their designs to address those needs. Nursing schools, in particular, experience evolving social, cultural, and environmental opportunities, making it impractical to rely on a single model for designing training programs. Therefore, nurse educators should familiarize themselves with diverse instructional design models to choose the most appropriate one for their classes and courses [10].

Second, in the development of our training program, we utilized the Purnell Model of Cultural Competence and Foronda's Cultural Humility Scale as two theoretical frameworks. This decision was supported by the findings of several studies that also employed the Purnell Model and Foronda's Cultural Humility Scale in their cultural care training programs [17, 21, 22]. There are several other established models that have been used as frameworks for curriculum development in cultural competence training. Some of these models include Leininger's Sunrise Model, Campinha-Bacote's Model of Cultural Competence [23, 24], Davidhizar & Giger's Transcultural Assessment Model [37], and the LEARN Model [38]. Brathwaite et al. (2005) emphasized the importance of incorporating cultural competence as a comprehensive framework for guiding research and educational interventions in healthcare [39]. This highlights the significance of integrating cultural competence into training programs to enhance the knowledge, skills, and attitudes of healthcare providers.

Third, in the step of evaluation, the results showed that the mobile app-based cultural care training program significantly improved the cultural capacity in the intervention group compared to the control group. The findings of our study align with previous research conducted in different countries, which evaluated the impact of training programs on the cultural competence of healthcare providers, including nursing students. While these studies did not utilize the cultural capacity scale, they employed various teaching methods [5, 21, 22, 35, 36]. One notable study by Sung and Park (2021) assessed a cultural competence training program for nurses using the Purnell model as the theoretical framework. The program was delivered through a mobile application in a group study format. The evaluation results demonstrated the effectiveness of the program in enhancing the cultural competence of nurses. The researchers suggested that mobile-based learning, facilitated by a structured model tailored to the specific subject matter, could be a cost-effective and efficient educational method for nursing education and in-service training [21].

Chang et al. (2017) conducted a study where they provided cultural competence training to health professionals through Facebook. The results of their 12-month follow-up study indicated that this training approach improved the cultural competence of health professionals. The findings suggested that Facebook could be utilized as a training delivery platform for providing personal and social learning, as well as cultural competency content in continuing education [36]. Similarly, Park et al. (2019) conducted a study that focused on a cultural nursing course. The course employed various teaching methods, including small group activities, discussions and presentations, experiential learning, reflective activities, and lectures. The results of the study demonstrated that this course significantly improved the cultural competence of the nursing students [35].

In a mixed-method study, researchers investigated the impact of a cultural competency course with field experience and a stand-alone cultural competence course on undergraduate nursing students. The quantitative phase of the study revealed that both educational methods significantly enhanced the cultural competence of nursing students when compared to the control group. However, there was no significant difference observed between the two teaching methods.

During the qualitative phase, the experiences of the stand-alone cultural competence course group yielded three main themes: journey to cultural competence, satisfaction of the cultural competence course, and suggestion for improvements. The students found the Purnell model to be an appropriate framework for cultural assessment in diverse patient populations. They expressed that the course had transformed their perspective on individuals from other cultures. They became familiar with the importance of empathy, sympathized with patients, respected their cultural beliefs, and accepted their health beliefs and behaviors. Additionally, some students were able to overcome their apprehensions regarding language barriers, different cultures, and feelings of unfamiliarity or limitation. As a result, they developed a positive outlook towards culturally diverse individuals [22].

Another mixed-method study examined the impact of two training methods, lecture, and lecture with standardized patient simulation, on the cultural competence of undergraduate nursing students. The results indicated that both training methods led to an increase in cultural competence among the students, with no significant difference observed between the two approaches. The qualitative findings of the study revealed that after undergoing lectures with standardized patient simulation, students experienced a decrease in anxiety when interacting with culturally diverse patients, and their comfort levels in patient interactions improved [5]. These findings align with similar studies conducted in Iran, which also demonstrated the effect of virtual cultural care training on the cultural competence of undergraduate students [10], postgraduate students [16], and nursing professors [26]. These studies suggest that given the current globalized world and the increasing prevalence of medical tourism, nursing and midwifery schools should prioritize cultural care education at all educational levels. Additionally, nurse educators should enhance their cultural competencies by participating in specialized empowerment programs.

In contrast to the present study, several studies have reported that cultural competence training programs did not yield a significant impact on overall cultural competence or specific areas such as participants' attitudes and skills. These findings highlight the importance of carefully planning and organizing cultural education programs with the aim of effectively enhancing the development of cultural competence [40–42]. Furthermore, Tosun et al. (2021) conducted a systematic review study that revealed a limited number of studies investigating the effectiveness of transcultural nursing education provided to nursing students. Among the 11 studies reviewed, 3 studies did not find any significant difference between the intervention and control groups. Additionally, the educational content, methods, and courses varied across the reviewed studies, lacking standardization. As a result, the researchers concluded that there was a need for more comprehensive, valid, and reliable measurement tools to evaluate the effectiveness of the training programs provided to nursing students [3].

Fourth, in the step of evaluation, the results also showed that the mobile app-based cultural care training program significantly improved cultural humility in the intervention group compared to the control, and the students were sometimes culturally humble. Our findings are consistent with the results of previous studies. Sánchez-Ojeda et al. (2021) demonstrated that completion of a cultural orientation training course led to a reduction or elimination of prejudiced and negative attitudes towards immigrants among nursing students [43]. Similarly, Johnson (2021) conducted a study on a cultural humility training program based on Fronda's rainbow model. The results showed positive effects on self-awareness, supportive interactions, cultural humility, and resilience of care providers. Additionally, all participants reported an increased awareness of personal biases and felt better prepared to handle challenging interactions with clients [17].

Another study by Ndiwane et al. (2017) incorporated cultural humility content into an advanced health assessment course for nursing students using simulation. The students expressed satisfaction with the training content and obtained the highest scores for the standardized patient scenarios after completing the objective structured clinical examination [44]. A study focused on exploring the experiences of nursing students engaging in global health service learning to practice cultural humility. The findings revealed that participating in health service-learning trips contributed to the development of cultural humility and the ability to establish supportive cross-cultural relationships. The process of cultural humility helped students overcome feelings of superiority and facilitated the formation of positive cross-cultural connections [29, 45].

In a systematic review conducted by Foronda et al. (2018) on cultural competence and cultural humility in simulation-based training, four themes emerged from the results related to learning from the simulation: cultural sensitivity and competence, insight, and understanding, communication, and confidence and comfort. However, none of the studies mentioned cultural humility specifically. The researchers emphasized that cultural humility in simulation-based education was not adequately addressed, indicating the need for modifications and changes in the educational content of simulation-based training [28].

The review of the aforementioned studies reveals both similarities and differences in the acquisition of cultural competence and humility across various contexts. Therefore, it is crucial to have a comprehensive understanding of the existing platform, needs, and strategies before implementing an effective intervention to enhance the cultural care competence of nursing students. Additionally, examining relevant literature can aid in designing comprehensive and efficient training programs. Nursing students acquire cultural competence, capacity, and humility through their experiences at university, during their studies, and through their work experiences. It is important to recognize that nursing education has the responsibility of preparing future nurses to develop cultural capacity and humility from their early student days. This process should continue through continuous on-the-job training, as nurses learn how to provide culturally diverse care with humility. Furthermore, the advancements in technology available today offer opportunities to enhance learning experiences for students through the use of modern communication tools.

## Limitations and strengths

The present study had several limitations that should be acknowledged. Firstly, the effectiveness of the mobile app-based cultural care training program in improving cultural capacity and humility was evaluated using self-report scales. Additionally, the study did not include a qualitative survey to explore nursing students' learning experiences and perspectives following the training program. Furthermore, due to time and financial constraints, data collection for the evaluation step was conducted over a one-month interval, which may not have captured long-term effects. Future research could benefit from employing mixed methods and conducting longitudinal studies to obtain more robust and comprehensive results.

Despite these limitations, the study also had notable strengths. The researchers had extensive experience in designing and evaluating a cultural care training program based on the ADDIE instructional model, as well as incorporating the Purnell Model for Cultural Competence and Foronda's Cultural Humility. This expertise can be valuable for developing future curricula aimed at fostering cultural capacity and humility among nursing students and other healthcare providers in different cultural contexts.

Furthermore, since the cultural capacity and humility questionnaire has been translated and culturally adapted for the first time in Iran, it presents an opportunity for researchers to utilize it in evaluating the cultural capacity and humility of nursing students at various stages of their education, as well as in different clinical and educational environments. Moreover, researchers can use it to test interventions aimed at enhancing cultural capacity and humility among nursing students.

## Conclusions

The study results indicated that the cultural care training program delivered through the mobile application had a positive impact on the cultural capacity and humility of undergraduate nursing students. Given the high cultural diversity in patients and clients seeking healthcare services, nursing education must prepare future nurses to provide culturally competent and humble care. Future nurses should be trained to recognize the values, prejudices, and assumptions of their multicultural societies and develop the necessary competencies to provide cultural care to clients from diverse cultural backgrounds. Nurse educators are committed to integrating the frameworks of cultural humility comprehensively into the theoretical and clinical courses of nursing students. They are actively exploring new methods to enhance these skills. Researchers also can contribute by conducting research to develop tools and measures for evaluating cultural issues in healthcare. Policymakers and authorities should recognize the importance of organizational cultural competence in healthcare. They are recommended to facilitate the provision of necessary resources and opportunities to establish organizational cultural competence in academic and clinical environments, aiming to foster cultural competence, capacity, and humility in nurses and nurse educators, who serve as role models for nursing students.

## Data Availability

The data are available upon request to the corresponding author after signing appropriate documents in line with ethical application and the decision of the Ethics Committee.

## Abbreviations

ADDIE: Analysis, design, development, implementation, and evaluation
I-CVI: Item Content Validity Index
S-CVI: Scale Content Validity Index
OSCE: Objective Structured Clinical Examination
CCS-A: The Cultural Capacity Scale Arabic

## References

[1] Ličen S, Prosen M (2023). The development of cultural competences in nursing students and their significance in shaping the future work environment: a pilot study. BMC Med Educ.

[2] Lin M-H, Wu C-Y, Hsu H-C (2019). Exploring the experiences of cultural competence among clinical nurses in Taiwan. Appl Nurs Res.

[3] Tosun B, Yava A, Dirgar E, Şahin EB, Yılmaz EB, Papp K, Tóthova V, Hellerova V, Prosen M, Licen S (2021). Addressing the effects of transcultural nursing education on nursing students’ cultural competence: a systematic review. Nurse Educ Pract.

[4] Lin C-J, Lee C-K, Huang M-C (2017). Cultural competence of healthcare providers: a systematic review of assessment instruments. J Nurs Res.

[5] Byrne D (2020). Evaluating cultural competence in undergraduate nursing students using standardized patients. Teach Learn Nurs.

[6] Zhang X, Zhou M (2019). Interventions to promote learners’ intercultural competence: a meta-analysis. Int J Intercult Relat.

[7] Asurakkody TA (2019). Predictors for transcultural self-efficacy of nursing students: Application of ecological model. Health Sci J.

[8] Bauer K, Bai Y (2018). Using a model to design activity-based educational experiences to improve cultural competency among graduate students. Pharmacy.

[9] Dunagan PB, Kimble LP, Gunby SS, Andrews MM (2014). Attitudes of prejudice as a predictor of cultural competence among baccalaureate nursing students. J Nurs Educ.

[10] Farokhzadian J, Nematollahi M, Dehghan Nayeri N, Faramarzpour M (2022). Using a model to design, implement, and evaluate a training program for improving cultural competence among undergraduate nursing students: a mixed methods study. BMC Nurs.

[11] Song M, Yang N (2016). Intercultural communicative competence, self-esteem cultural competence of nursing students. Int J Adv Nurs Educ Res.

[12] Almutairi AF, Adlan AA, Nasim M (2017). Perceptions of the critical cultural competence of registered nurses in Canada. BMC Nurs.

[13] Nematollahi M, Farokhzadian J, Nayeri ND, Darban F, Faramarzpour M (2022). Explaining the educational challenges in the path of cultural competence: The experiences of Iranian nursing students. J Prof Nurs.

[14] Markey K, Prosen M, Martin E, Repo Jamal H (2021). Fostering an ethos of cultural humility development in nurturing inclusiveness and effective intercultural team working. J Nurs Manag.

[15] Firoozi F, Mozaffari N, Iranpour S, Molaei B, Shamshiri M (2020). The status of cultural care among nurses working in different wards of teaching hospitals in Ardabil, Iran: a cross-sectional survey study. Int J Care Coord.

[16] Fadaeinia MM, Miri S, Azizzadeh Forouzi M, Roy C, Farokhzadian J. Improving cultural competence and self-efficacy among postgraduate nursing students: results of an Online Cultural Care Training Program. J Transcult Nurs. 2022;33(5):642–51. 10.1177/10436596221101925 35684956

[17] Johnson C. Reducing implicit bias: evaluating cultural humility and mindfulness practices in the perinatal microsystem. Master's Projects and Capstones. 1259. University of San Francisco; 2021. https://repository.usfca.edu/capstone/1259.

[18] Khachian A, Zarei MR, Haghani H, Khani F (2020). The correlation between the cultural competence of nurses with their care behaviors in the teaching health centers affiliated to iran university of medical sciences. Iran J Nurs.

[19] Choi J-S, Kim J-S (2018). Effects of cultural education and cultural experiences on the cultural competence among undergraduate nursing students. Nurse Educ Pract.

[20] Torkaman M, Sabzi A, Farokhzadian J (2022). The effect of patient safety education on undergraduate nursing students’ patient safety competencies. Commun Health Equity Res Policy.

[21] Sung S, Park H-A (2021). Effect of a mobile app-based cultural competence training program for nurses: A pre-and posttest design. Nurse Educ Today.

[22] Ho T-T-T, Oh J (2022). Development and Evaluation of Cultural Competence Course on Undergraduate Nursing Students in Vietnam. Int J Environ Res Public Health..

[23] Noble A, Nuszen E, Rom M, Noble LM (2014). The effect of a cultural competence educational intervention for first-year nursing students in Israel. J Transcult Nurs.

[24] Kohlbry PW (2016). The impact of international service-learning on nursing students’ cultural competency. J Nurs Scholarsh.

[25] Chang C-Y, Lai C-L, Hwang G-J (2018). Trends and research issues of mobile learning studies in nursing education: a review of academic publications from 1971 to 2016. Comput Educ.

[26] Rahimi M, Khodabandeh Shahraki S, Fatehi F, Farokhzadian J (2023). A virtual training program for improving cultural competence among academic nurse educators. BMC Med Educ.

[27] Purnell L: Transcultural health care: A culturally competent approach: FA Davis. Appendices Declaration of Interest The author has declared the conflicts of interest below Dr Petra Verdonk declares a conflict of interest as a current guest theme co-editor of" Diversity in Medical Education” theme in AMEE MedEdPublish There are no other conflicts of interest to declare. 2012.

[28] Foronda CL, Baptiste D-L, Pfaff T, Velez R, Reinholdt M, Sanchez M, Hudson KW (2018). Cultural competency and cultural humility in simulation-based education: an integrative review. Clin Simul Nurs.

[29] Matthew S, Hockett E, Samek L (2018). Learning cultural humility through stories and global service-learning. J Christ Nurs.

[30] Perng SJ, Watson R (2012). Construct validation of the nurse cultural competence scale: a hierarchy of abilities. J Clin Nurs.

[31] Cruz J, Machuca Contreras F, Ortiz López JE, Zapata Aqueveque C, Vitorino L (2018). Psychometric assessment of the cultural capacity scale Spanish version in Chilean nursing students. Int Nurs Rev.

[32] Cruz JP, Colet PC, Bashtawi MA, Mesde JH, Cruz CP (2017). Psychometric evaluation of the cultural capacity scale arabic version for nursing students. Contemp Nurse.

[33] Foronda C, Porter A, Phitwong A (2021). Psychometric testing of an instrument to measure cultural humility. J Transcult Nurs.

[34] Bhat AM, Wehbe-Alamah H, McFarland M, Filter M, Keiser M (2015). Advancing cultural assessments in palliative care using web-based education. J Hosp Palliat Nurs.

[35] Park HS, Jang HJ, Jeong GH (2019). Effects of a cultural nursing course to enhance the cultural competence of nursing students in Korea. J Educ Eval Health Prof.

[36] Chang L-C, Guo JL, Lin H-L (2017). Cultural competence education for health professionals from pre-graduation to licensure delivered using facebook: twelve-month follow-up on a randomized control trial. Nurse Educ Today.

[37] Smit EM, Tremethick MJ (2013). Development of an international interdisciplinary course: a strategy to promote cultural competence and collaboration. Nurse Educ Pract.

[38] Berlin A, Nilsson G, Törnkvist L (2010). Cultural competence among Swedish child health nurses after specific training: a randomized trial. Nurs Health Sci.

[39] Brathwaite AE (2005). Evaluation of a cultural competence course. J Transcult Nurs.

[40] Majda A, Zalewska-Puchała J, Bodys-Cupak I, Kurowska A, Barzykowski K (2021). Evaluating the effectiveness of cultural education training: cultural competence and cultural intelligence development among nursing students. Int J Environ Res Public Health.

[41] Kula Y, Cohen O, Clempert N, Grinstein-Cohen O, Slobodin O (2021). Educating nursing students for cultural competence in emergencies: a randomized controlled trial. BMC Nurs.

[42] Saffee CL. Improving the cultural competency of registered nurses in a bachelor’s completion program. Doctoral dissertation. Capella University; 2015.

[43] Sánchez-Ojeda MA, Fernández-Gomez E, Ortiz-Gómez MdM, Alemany-Arrebola I (2021). The influence of training in cross-culturalism on future nurses: can education change prejudiced attitudes toward migrants?. J Transcult Nurs.

[44] Ndiwane AN, Baker NC, Makosky A, Reidy P, Guarino AJ (2017). Use of simulation to integrate cultural humility into advanced health assessment for nurse practitioner students. J Nurs Educ.

[45] Sedgwick A, Atthill S (2020). Nursing student engagement in cultural humility through global health service learning: an interpretive phenomenological approach. J Transcult Nurs.

